# Energy Saving in Data Processing and Communication Systems

**DOI:** 10.1155/2014/452863

**Published:** 2014-06-29

**Authors:** Giuseppe Iazeolla, Alessandra Pieroni

**Affiliations:** Department of Enterprise Strategy and Applied Technology, “Guglielmo Marconi” University of Study, Roma, Italy

## Abstract

The power management of ICT systems, that is, data processing (Dp) and telecommunication (Tlc) systems, is becoming a relevant problem in economical terms. Dp systems totalize millions of servers and associated subsystems (processors, monitors, storage devices, etc.) all over the world that need to be electrically powered. Dp systems are also used in the government of Tlc systems, which, besides requiring Dp electrical power, also require *Tlc-specific* power, both for *mobile* networks (with their cell-phone towers and associated subsystems: base stations, subscriber stations, switching nodes, etc.) and for *wired* networks (with their routers, gateways, switches, etc.). ICT research is thus expected to investigate into methods to reduce Dp- and Tlc-specific power consumption. However, saving power may turn into waste of performance, in other words, into waste of ICT quality of service (QoS). This paper investigates the Dp and Tlc power management policies that look at compromises between power saving and QoS.

## 1. Introduction

The growth in ICT energy consumption is driven by the growth of demand for greater data processing (Dp) and larger access to telecommunications (Tlc), within almost every organization.

This growth has a number of important implications, including [1]

- increased energy costs for business and government,
- increased emissions, including greenhouse gases, from electricity generation,
- increased strain on the existing power grid to meet the increased electricity demand,
- increased capital costs for expansion of data center capacity and construction of new data centers,
- increased capital costs for expansion of wired and wireless access to communications.

Making 100 the total electrical power consumption for the ICT, around 14% is taken by the mobile Tlc, 74% by the wired Tlc, and the remaining 12% by the Dp technology.

Dp, however, is only apparently the less powered sector, since Tlc is itself is a Dp consumer, and so any effort to reduce the Dp power consumption may produce cascade effects that also reduce the Tlc one. Studying ways to save Dp power is thus central to any study for ICT power control and optimization.

In the US, power absorbed by data centers is estimated in more than 100 billion kW, for an expenditure of $ 8 billion a year that corresponds to the expenditure in electricity of about 17 million homes [2].

This same US-local data-center problem becomes a global one when seeing power consumption by web companies, say Google, Yahoo, and so forth. The number of Google servers will reach an estimated 2,376,640 units by the end of 2013 [3].

Assuming a busy server absorbs around 240 W of power, Google will need about 600 MW of electrical power by the end of year 2013.

In Tlc systems, about 90% of Tlc-specific power consumption is concentrated in the routers. The links only absorb 10%. Current routers consume between 0.01 and 0.1 W/Mbps [4].

IT research is thus expected to investigate methods to reduce power absorbed by Dp and Tlc systems. To do that, one may decide to adopt policies to periodically switch off Dp servers or Tlc routers when they are in an idle state.

Such policies, however, are to be sufficiently intelligent not to degrade the system quality of service (QoS). Indeed, returning an off server or an off router to its on state requires spending a nonnegligible amount of setup time that makes the server or router slower to respond to customer requests. This may turn into low-quality services such as low response to web queries and unsatisfactory VoIP communications and streaming of data. Any research in power management should thus look at compromises between power saving and QoS.

In this paper, Section 2 studies Dp power management policies and Section 3 studies Tlc power management policies.

## 2. Power Management in Dp Systems

Data centers have become common and essential to the functioning of business, communications, academic, and governmental systems.

During the past years, increasing demand for Dp services has led to significant growth in the number of data centers, along with an estimated doubling in the energy used by servers and the power and cooling infrastructure that supports them.

**Figure 1:**
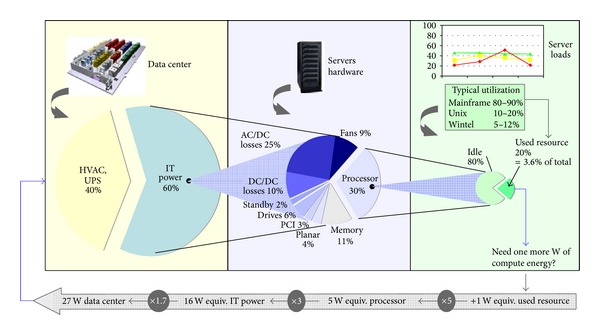
Data Ccnter energy consumption sources [25].

Figure 1 illustrates the way energy is spent in data centers. Heating and services for ventilation and air conditioning/backup (HVAC/UPS) absorb around 40% of electrical energy and Dp services the remaining 60%. The latter is in turn divided between AC/DC losses (25%), DC/DC losses (10%), fans, drives, PCI, and so forth, and memory consumptions (for a total 35%) and the remaining 30% is consumption in server processors.

In other words, the processors consumption totalizes 0.30 × 0.60, that is, 20% of total data center consumption. Such an amount, even though apparently negligible with respect to the total, is the main cause of the remaining 80%. Thus, any effort to reduce the processors 20% may produce cascade effects that also reduce the remaining 80%.

**Figure 2:**
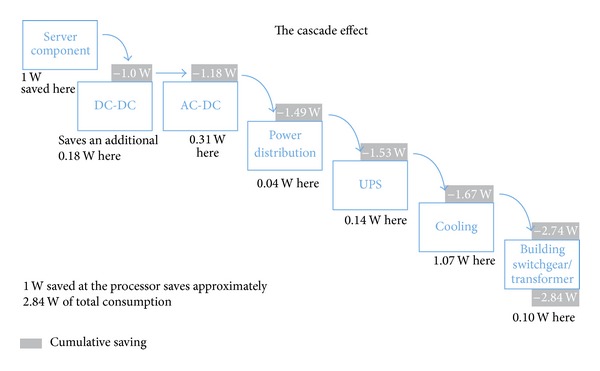
Cascade effect of energy saving in data centers [26].

Figure 2 shows that 1 W savings at servers component level (processor, memory, hard disk, etc.) create a reduction in data center energy consumption of approximately 2.84 W.

For this reason, any research in ICT power saving should concentrate on policy to reduce Dp consumption at server components level.

Data centers can be seen as composed of a number of servers that can be organized into single farms or multifarms. In the following, Section 2.1 sees power saving policies in the single-farm case and Section 2.2 at the multifarm one.

### 2.1. Energy Saving in* Single-Farm* Data Centers

Most of power absorbed by the servers of a farm is wasted, since servers are busy (i.e., making processing work) only 20% to 30% of the time, on average. So, energy saving requires the adoption of management policies to avoid powering the servers when they are not processing. In other words, policies decide in which state (idle or off) to keep the servers when not busy. Two types of server management policies will be considered: static and dynamic policies.

#### 2.1.1. Energy Saving with Static Policies

One may assume that a busy server in the on state absorbs around 240 W (*P*_ON_), an idle server about 160 W (*P*_IDLE_), and an off server 0 W (*P*_OFF_). So why not keep in the idle state or in the off state the servers when not busy? Just since switching a server from off to on consumes a time overhead. Thus, a power-saving policy may result in a time-wasting problem. As a consequence, the servers may lose performance (e.g., increased response time to the incoming jobs, lower throughput of communication packets, etc.) and its service may become unacceptable to customers.

To turn on an off server, we must first put the server in setup mode. During the setup period, the server cannot process jobs. The time spent in setup is called setup time. In [5] the authors consider server farms with a setup cost. Setup costs always take the form of a time delay, and sometimes there is also a power penalty, since during that entire period the server consumes the same power as being in the on state.

In [5] three different policies are studied to manage server farms: On/Idle policy, On/Off policy, and On/Off/Stag policy.

Under the On/Idle policy, servers are never turned off. All servers are either on or idle and remain in the idle mode when there are no jobs to serve. Assume that the farm consists of *n* servers; if an arrival finds a server idle, it starts serving on the idle server. Arrival that finds all *n* servers on busy, joins a central queue from which the servers pick jobs when they become idle.

The On/Off policy consists instead of immediately turning off the servers when not in use. As said above, however, there is a setup cost (in terms of time delay and of additional power penalty) for turning on an off server.

Finally, the On/Off/Stag policy is the same as the On/Off one, except that at most 1 server can be in setup at any point of time. This policy is known as the “staggered boot up” policy in data centers or “staggered spin up” in disk farms [5–7].

**Figure 3:**
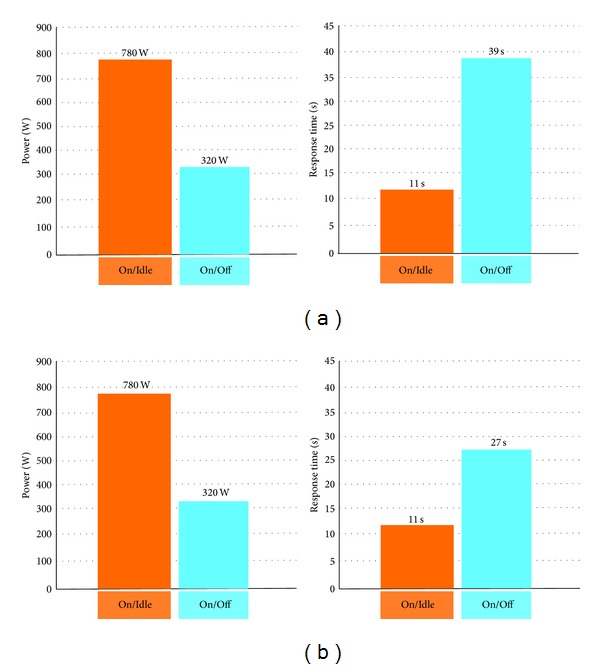
(a) Experimental results with 4 servers, utilization = 30% average setup time = 200 sec; average job size (service time) = 7 sec [2]. (b) Experimental results with same parameters of (a) except for SPTF or SJF queueing discipline.

Figure 3(a) compares the On/Off and On/Idle policies for an example case.

The On/Idle policy proves to be better in terms of response time, because the incoming jobs do not suffer from setup time delays but involves a larger amount of power waste with respect to the On/Off policy, because of the amount of power an idle server absorbs.

**Figure 4:**
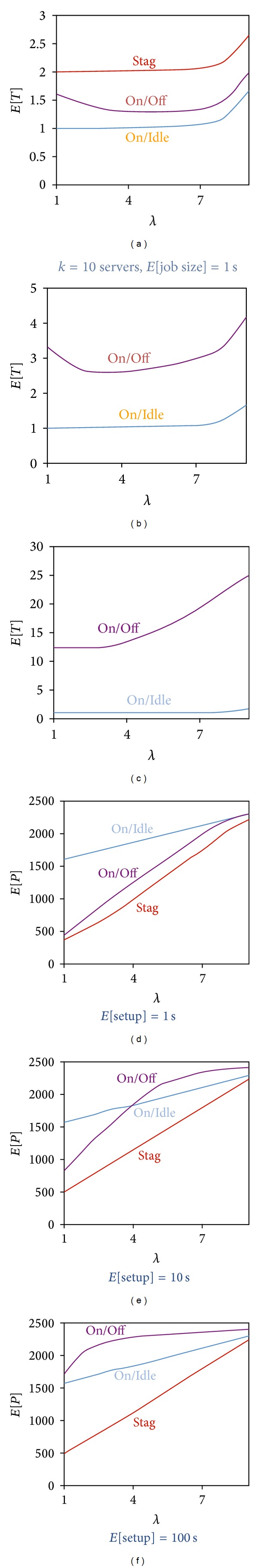
Effects of server management policies and setup time on response time and power consumption [2].

Figure 4 compares the three server management policies in a farm consisting of *k* = 10 servers, when the average setup time changes from 1 to 100 sec and the average processing load *λ* (i.e., average job arrival rate) from 1 to 7 job/sec. The mean job size (service time) is assumed to be 1 sec.

Comparison is on the basis of the resulting mean response time *E*[*T*] to the incoming jobs and the average power consumption *E*[*P*].

In the On/Idle case, when *λ* is low, there is no waiting and thus the mean response time *E*[*T*] is of about the mean job service time (1 sec) and increases for increasing *λ*.

A similar trend can be observed for the On/Off/Stag policy, since

(1) $$\begin{array}{c} E{\left[T\right]}_{\text{ON}/\text{OFF}/\text{STAG}}=E{\left[T\right]}_{\text{ON}/\text{IDLE}}+E\left[\text{setup}\;\text{time}\right], \end{array}$$

as shown in [5].

For the On/Off policy, instead, the response time curve follows a bathtub behavior.

When the load *λ* is low, the mean response time is high, since almost every arrival finds servers in the off state, and thus every job incurs the setup time. For medium loads, servers are less frequently switched to the off condition and thus jobs are more likely served by available servers in the on state and do not incur in setup times. For high loads, finally, the mean response time increases due to large queueing in the system.

For the power consumption, one can show [5] that *E*[*P*]_ON/OFF/STAG_ < *E*[*P*]_ON/OFF_, since at most one server can be in setup for the On/Off/Stag policy. There also results *E*[*P*]_ON/OFF_ < *E*[*P*]_ON/IDLE_, since servers are turned off in the On/Off case. However, for loads *λ* above the medium, there results *E*[*P*]_ON/OFF_ > *E*[*P*]_ON/IDLE_ for medium setup time; that is, *E*[setup  time] = 10 sec, while for large setup time, that is, *E*[setup  time] = 100 sec, there always results *E*[*P*]_ON/OFF_ > *E*[*P*]_ON/IDLE_, because of the large amount of power wasted in turning servers on in the On/Off policy.

Table 1 gives a synthetic comparison of the three considered policies in terms of response time and power consumption.

In conclusion, any reduction in power consumption is paid by an increase in response times. So, why not adopt queueing disciplines that minimize average response times? The SPTF (shortest processing time first) [8] or SJF (shortest job first) [9] queueing discipline is known to perform better than the common FIFO (first in first out). Its use can then reduce the amount to pay in terms of response time to obtain a given power saving.

Figure 3(a) (that illustrates the FIFO queueing case) shows that to reduce the power consumption from 780 to 320 W we have to pay an increase from 11 to 39 sec in average response time.

Figure 3(b) illustrates that if the SPTF discipline is used instead; the debt to pay in response time is much smaller (from 39 to 27 sec) as proved in our simulations studies [10].

#### 2.1.2. Seeking the Optimal (*π*, *δ*) Strategy for the Single-Farm Data Centers

Under the assumption of Poisson arrivals, exponential service times, and deterministic setup times, authors in [11] prove that the optimal, or nearly optimal, combination of (*π*, *δ*), with *π* being one policy from the set {On/Off, On/Idle} and *δ* one queueing discipline from the set {FIFO, LIFO, RAND}, means minimizing a new metric called ERP (energy-response time product) and is defined as

(2) $$\begin{array}{c} \text{ERP}\left(\pi ,\delta \right)=E\left[P\right]\left(\pi ,\delta \right)\times E\left[T\right]\left(\pi ,\delta \right). \end{array}$$

Minimizing ERP(*π*, *δ*) can be seen as maximizing the performance per Watt, with performance defined as the inverse of the mean response time [11].

In other words, according to their results, there is no need to consider other policies than the On/Off and the On/Idle policies.

They, however, only study the effects of moving from one policy *π* to another, without paying attention to the effects of also moving from a *δ* = FIFO discipline to another time-independent discipline.

Under the FIFO assumption, however, they find that the On/Idle policy is typically superior to the remaining two in terms of ERP (*π*, *δ*).

Our aim is to extend such results by studying the effects of the queueing discipline *δ*, both on the ERP(*π*, *δ*) index and on the *E*[*P*](*π*, *δ*) and *E*[*T*](*π*, *δ*) indices separately.

More precisely, the following four (*π*, *δ*) strategies are investigated in the paper:

1. (On/Idle, FIFO),
2. (On/Idle, SPTF),
3. (On/Off, FIFO),
4. (On/Off, SPTF).

And for each of such strategy the ERP(*π*, *δ*) product is studied besides the *E*[*P*](*π*, *δ*) and *E*[*T*](*π*, *δ*) indices. Two largely different setup times (*E*[setup] = 1 s and *E*[setup] = 100 s) will be used to stress the effect of the setup time on the On/Idle and On/Off polices.

Similarly, two largely different farm data center loads, low *ρ* (*ρ* ≤ 0.5) and high *ρ* (*ρ* → 1), will be used to stress the effect of the queueing disciplines.

The following farm data center characteristics are assumed: server mean setup time *E*[setup] = 1 sec (or 100 sec), server *P*_ON_ = 240 W, server *P*_SETUP_ = 240 W, server *P*_IDLE_ = 150 W, server *P*_OFF_ = 0 W, mean job service time *S* = 1 sec, and *n* = 30 servers.

Table 2 shows simulation results [10] that compare the Dp power and QoS indices in the low setup case (*E*[*T*setup] = 1 sec).

- Seeing at the power consumption *E*[*P*], we note that there is no effect by the queueing discipline *δ* on the power consumption *E*[*P*], while there is an effect by the policy *π* for low *ρ*. Indeed, a drastic reduction can be seen (from 6000 W to 4200 W, for low *ρ*) when moving from On/Idle to On/Off, since when *ρ* is low, the waiting queue is almost empty and thus a large number of servers is in the off state. For high *ρ* instead, the power consumption *E*[*P*] remains unchanged (*E*[*P*] = 7100 W) with the discipline *δ*, since the queue is always full and thus the servers remain always in the on state.
- Seeing at the response time *E*[*T*], we note that there is an effect both by the queueing discipline *δ* and by the policy *π*. The effects hold both for low *ρ* and for high *ρ*. In the On/Idle case, when *ρ* is low, there is no waiting in the Dp queue and thus the mean response time *T* is about the mean job service time (*S* = 1 sec), while it increases (*E*[*T*] = 1.8 sec for *δ* = FIFO and *E*[*T*] = 1.3 sec for *δ* = SPTF) for high *ρ*.
- In the On/Off case, when *ρ* is low, the mean response time is higher (*E*[*T*] = 1.2 sec with no effect by the discipline; the queue is empty), since almost every arrival finds servers in the off state, and thus every job incurs in the setup time. For high *ρ*, instead, the mean response time increases (*E*[*T*] = 2 sec for *δ* = FIFO and *E*[*T*] = 1.35 sec for *δ* = SPTF) due to large queueing. As predicted above, we can see that the benefit in response time one may obtain from moving FIFO to SPTF is larger than the one obtainable from moving On/Off to On/Idle. Indeed (see high *ρ*) moving from the (On/Off, FIFO) strategy to the (On/Idle, FIFO), the response time *E*[*T*] changes from 2 to 1.8 (a 10% reduction). Moving, instead, from the (On/Idle, FIFO) strategy to the (On/Idle, SPTF) the response time *E*[*T*] changes from 1.8 to 1.3 (an almost 30% reduction).
- Seeing at the ERP (*π*, *δ*) index, its values are a consequence of the *E*[*P*] and the *E*[*T*] ones. Table 2 shows that the optimal ERP(*π*, *δ*) is obtained for the (On/Off, FIFO) strategy and for the (On/Off, SPTF) strategy when *ρ* is low, while it is obtained for the (On/Idle, SPTF) strategy only when *ρ* is high.

For the high setup time case (*E*[setup] = 100 sec), the reader is sent to [12].

In summary, making predictions of the Dp power management policies that optimizes

- the Dp power consumption (minimum absorbed Watts) or
- the Dp performance (minimum response_time) or
- the Dp performance-per-Watt (minimum response_time-per-Watt)

is a nontrivial task. The most significant policies *π* are first to be drawn from the universe of all possible policies. Then, for each such policy, the effects of time-dependent and time-independent queueing disciplines are to be studied. On the other hand, once the modeling work has been done, the work the server-farm manager has to perform to direct his Dp is greatly simplified, since the universe of all possible (*π*, *δ*) strategies he needs to choose from is drastically reduced to very small set of most significant strategies.

#### 2.1.3. Energy Saving with Dynamic Policies

In any practical situation, the load *λ* changes over time, according to a given pattern *λ*(*t*). One should then find policies that adapt themselves to changing load patterns. This is not the case of the policies introduced in Section 2.1.1, which are somehow static in nature and remain efficient only for given values of *λ*, while becoming inefficient for other values. Looking, for example, at Figure 4 case with *E*[setup] = 10 sec, one can see that for changing values of *λ* there are situations in which the On/Off policy consumes less power than On/Idle and vice versa.

For this reason, two adaptive versions of the On/Off and On/Idle policies are known in the literature, respectively, called DelayedOff and LookAhead, which dynamically adapt themselves to changing loads [11].

The DelayedOff policy is an improvement of the On/Off. According to DelayedOff, when a server goes idle, rather than turning off immediately, it sets a timer of duration *t*_wait_ and sits in the idle state for *t*_wait_ seconds. If a request arrives at the server during these *t*_wait_ seconds, the server goes back to the busy state (with zero setup cost); otherwise, the server is turned off.

The LookAhead policy is an improvement of the On/Idle. Under such a policy, the system fixes an optimally chosen number n* of servers maintained in the on or idle states. According to the standard On/Idle, if an arrival finds a server idle it starts serving on the idle server. Arrivals that find all n* server on busy, join a central queue from which servers pick jobs when they become idle.

The optimal *t*_wait_ and the optimal n* of the two policies, respectively, are chosen to minimize the ERP index. As said above, minimizing ERP can be seen as maximizing the performance per Watt, with performance defined as the inverse of the mean response time [11].

In the LookAhead policy, n* changes as a function of time. Indeed, the policy calculates n*(*t*) for each time t basing on the forecast of the load *l*(*t*) at time *t*.

**Figure 5:**
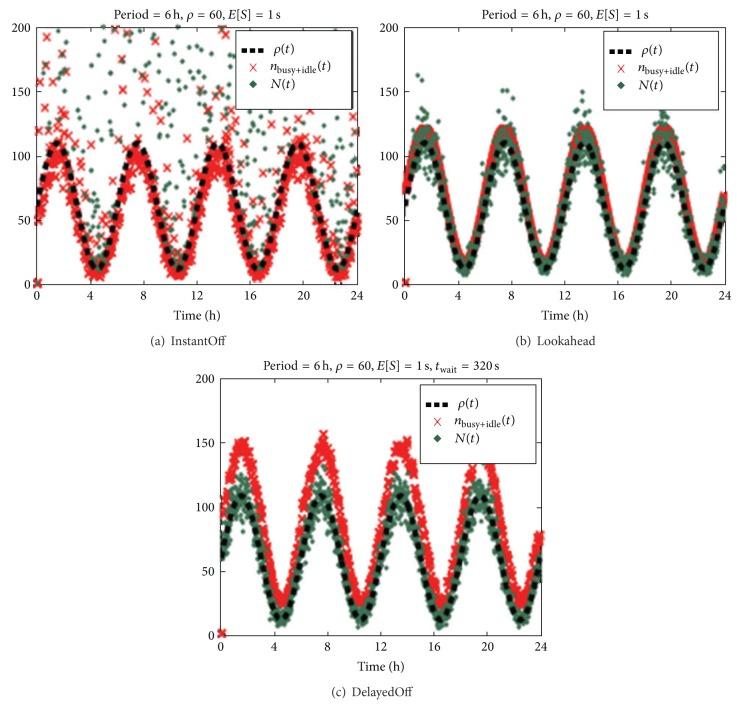
Effects of dynamic policies with respect to the static On/Off [11].

Figure 5 illustrates the autoscaling capabilities of the LookAhead and DelayedOff policies [11], with respect to the conventional On/Off, for Poisson arrivals with *l*(*t*) changing sinusoidally with time (period = 6 hrs).

Figure 5(a) refers to the* On/Off* policy (called* InstantOff*), Figure 5(b) to the* LookAhead* and Figure 5(c) to the* DelayedOff.* The dashed line denotes the varying load at time *t*, *λ*(*t*). The crosses denote the number *n*_busy+idle_(*t*) of servers that are busy or idle at time *t*, and the dots denote the number *N*(*t*) of jobs in the system at time *t*.

The illustration shows how, with the two dynamic policies, number *n*_busy+idle_(*t*) and number *N*(*t*) almost completely follow the behavior of the demand pattern *λ*(*t*), while in the On/Off case such numbers are somewhat dispersed; in other words, some servers remain in the idle state whereas they should be busy and vice versa, with the consequence of waste of power and worsened response time.

The two dynamic policies above simply try to optimize the *E*[*P*] by *E*[*T*] product.

In many practical situations, instead, the objective is to meet a given average response time, according to requirements dictated by specific service level agreements (SLAs).

In this case, specific dynamic policies have been introduced, which try to respect the *E*[*T*] requirement while minimizing the average power consumed by the servers (*P*_avg_) and the average number of used servers (*N*_avg_).

Such policies are known as the AutoScale policy [13], the AlwaysOn policy [14], the Reactive policy [15], and the Predictive MWA policy [16–19]. The latter will not be dealt with here, and we will only treat the AutoScale policy, which is an evolution of the remaining three.

The AutoScale policy generalizes the use of the *t*_wait_ time already seen for the DelayedOff policy. Differently from this latter, however, is that in the AutoScale case each server decides autonomously when to turn off, setting a timer of duration *t*_wait_ and sitting in the idle state for *t*_wait_ sec. As with the DelayedOff, however, if a request arrives at the server during these *t*_wait_ sec, then the server goes back to the busy state (with zero setup cost). Otherwise, the server is turned off.

**Figure 6:**
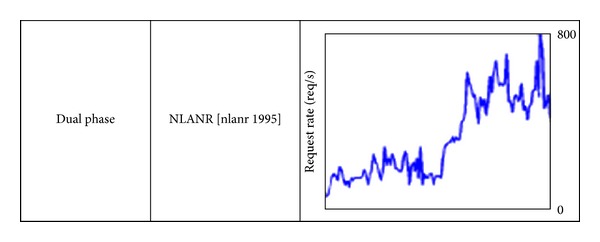
Dual phase pattern [13].

The AutoScale and the three remaining policies have been evaluated in [13] according to a specific load pattern *λ*(*t*) varying over time between 0 and 800 req/s (see Figure 6). Such a pattern, known as dual phase pattern is used to represent the diurnal nature of typical data center traffic, where the request rate is low at the night time and high at day time.

**Figure 7:**
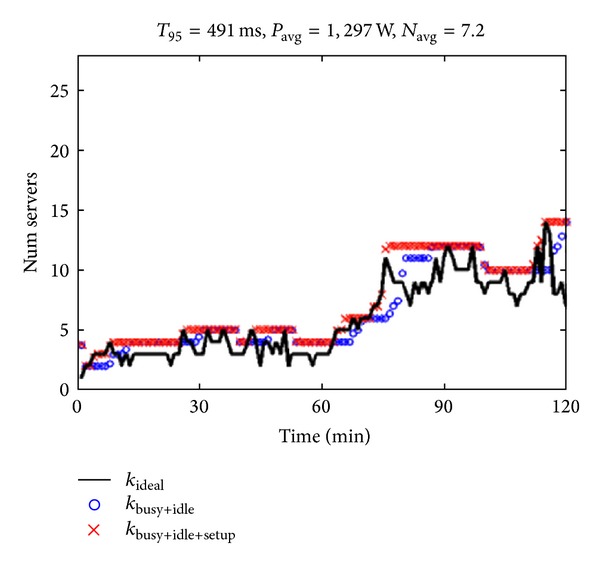
Effects of dynamic AutoScale policy [13].

Figure 7 illustrates the performance of the AutoScale policy, when the time requirement to meet is a 95-percentile response time *T* goal of 400 to 500 ms (denoted* T*_95_). In the illustration, the red lines denote the number *k*_busy+idle+setup_(*t*) of busy+idle+setup servers and the blue lines the number *k*_busy+idle_(*t*) of busy+idle servers at time *t*. The *k*_ideal_ line represents the number of servers that should be on at any given time to fully satisfy the demand *λ*(*t*).

The illustration shows how, with the AutoScale policy, there is no dispersion in the available servers and the number *k*_busy+idle+setup_(*t*) and number *k*_busy+idle_(*t*) almost totally follow the demand pattern *λ*(*t*), and a* T*_95_ = 491 ms goal is achieved, with *P*_avg_ = 1,297 W and *N*_avg_ = 7.2 servers.

In the mentioned similar policies (AlwaysOn, Reactive, and Predictive MWA), instead, the* T*_95_ requirement can be seen to be met only at the expense of server dispersion and/or at the expense of *P*_avg_ and *N*_avg_ [13].

Indeed, in the AlwaysOn case the* T*_95_ requirement is met (*T*_95_ = 291 ms) but at the expense of a large dispersion in the available servers and large power consumption (*P*_avg_ = 2,322 W and *N*_avg_ = 14).

In the Reactive case, instead, a low dispersion of servers is achieved, with low power and low number of servers (*P*_avg_ = 1,281 W, *N*_avg_ = 6.2), but the time requirement is absolutely out of range (*T*_95_ = 11,003 ms).

A better time performance (*T*_95_ = 7,740 ms) is found in the Predictive MWA with similarly low dispersion of servers and similarly low power and number of servers (*P*_avg_ = 1,276 W, *N*_avg_ = 6.3).

### 2.2. Energy Saving in* Multifarm* Data Centers

Energy saving in multifarms is based on so-called self-organization and self-differentiation algorithms, whose goal is to transfer the load from a server to a less loaded one, to maximize the power efficiency of the whole data center.

These algorithms are widely adopted in the autonomic computing field. The term autonomic indicates systems able to self-manage, self-configure, self-protect, and self-repair; thus, systems have no need of external action to be managed [20].

**Figure 8:**
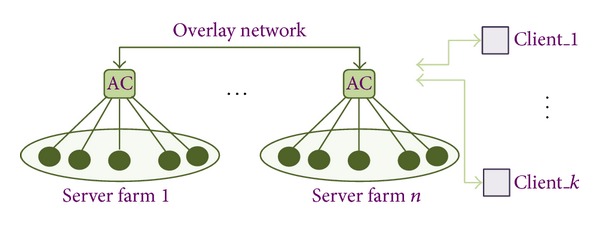
Example of a multifarm data center.

Figure 8 illustrates the typical multifarm architecture that consists of a series of server farms (1 through *n*) each farm controlled by a so-called autonomic component (AC), with the ACs interacting through an overlay network (ON). Each farm serves a number of clients (1 trough *k*).

The ON is a self-organized network, in other words a network which is created, maintained, and optimized through self-organization algorithms which cluster the ACs according to their properties or type [21].

The ACs, in turn, execute a particular kind of self-organization algorithm called self-differentiation algorithm, which takes decentralized decisions on the state and configuration of the ACs.

The AC aims to put state the servers in idle and transfer the load on the other servers to limit performance degradation. Three types of self-differentiation algorithms are known: Stand-by, Load Distribution, and Wake-up algorithms whose details can be found in [21].

The algorithms were evaluated by means of simulations of a use-case in which server farms are in charge to serve requests issued by a set of clients. Each client performs several requests, before terminating the connection. The percentage of energy that can be saved in a day goes from about 7% to about 12%, with a debt to pay in terms of response time from about 9 units of time (when the power saving is 7%) to about 11 units of time (when the power saving is 12%).

## 3. Energy Management in Tlc Systems

Tlc systems may consist of wired or wireless access networks or of a combination thereof.

In addition to the basic Dp infrastructure, Tlc systems also include Tlc-specific subsystems: cell-phone, towers with associated base stations, subscriber stations, switching nodes, and so forth, for the wireless part, and communication processors, routers, gateways, switches, and so forth, for the wired part.

Power management in Tlc systems, thus, includes not only power optimization of their Dp infrastructure, but also power optimization of Tlc-specific subsystems.

In this section we will only deal with Tlc-specific subsystems, since the power optimization of Dp infrastructure is dealt with is already seen in Section 2.

**Figure 9:**
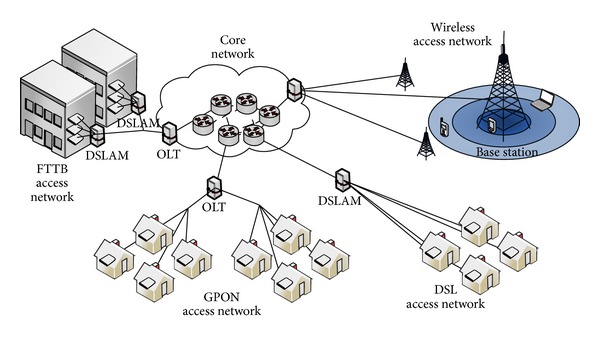
Typical Tlc network architecture [4].

Figure 9 describes a typical Tlc architecture, which combines wired and wireless communication networks.

In the wired part, three main types of connections are found: (1) the twisted pair copper cable connection based on the DSL (digital subscriber line) technology; (2) the coax cable connection based on the DOCS (data over cable service) technology; and (3) the optical fiber connection based on the GPON (gigabit passive optical network) technology, used when higher bit rates are required. The illustration also shows the DSLAM (DSL access multiplexer) nodes, the OLT (optical line termination) nodes, and the FTTB (fiber to the building) nodes.

In order to interconnect different user areas, a core network is used, that consists of a number of core nodes that are interconnected through wavelength-division multiplexed (WDM) optical fiber links, usually in a mesh or ring topology.

In the wireless part of the network we find base stations (BS) to which the user's devices are connected by means of radio signals. Each BS is further connected to the core network through a so-called backhaul network. Different technologies can be found, from WiMAX (worldwide interoperability for microwave access) [22], to HSPA (high speed packet access), and to the most recent LTE (long term evolution).

In such a system, about 90% of Tlc-specific power consumption is concentrated in the routers (with 75% the line cards, 10% the power supply and fans, and 10% the switch fabric) [23]. Current routers consume between 0.01 and 0.1 W/Mbps. One can calculate that at ADSL access rates (8 Mbps) the power absorbed per subscriber is of about 0.24 W/subs, while at 100 Mbps becomes of about 3 W/subs [4].

Currently, Tlc networks are designed to handle the peak loads. Designing adaptable networks, where one can switch off line cards when the demand is lower and can lead to lower power consuming networks.

In core networks this can be achieved by use of dynamic topology optimization algorithms: from all possible topologies that satisfy the required traffic demand, the topologies with lower overall power consumption are chosen. By such algorithms, reductions of power consumption for more than 50% during off-peak hours can be achieved [24].

Base stations (BS) with differentiated cell sizes are the key in wireless networks optimization if the so-called hybrid hierarchical BS deployment is used.

A low layer access network is first created, providing a low bit rate (but large cell sizes) to the users. In the higher layers, BS with higher bit rates (but smaller cell sizes) is utilized to provide high bandwidth connections when required. The advantage is that the higher layers can be switched to the idle and only switched on with high traffic demand.

Tlc power optimization also tries to minimize the power consumption of the home gateways. These are individual devices that only need to be on when the user is active. At other times, they could be switched off. In reality this is rarely operated, but legislations concerning standby power consumption standards of 0.5 W are emerging [4].

## 4. Conclusions

The power management of ICT systems, that is, data processing (Dp) and telecommunication (Tlc) systems, is a complex issue with implications in economical terms.

The paper has illustrated methods to optimize Dp power consumption by use of power management policies (static and dynamic policies) that yield electrical power saving while maintaining the system QoS at acceptable levels.

The paper has also illustrated methods to optimize Tlc power consumption by use of power management policies to be adopted in wired and wireless Tlc systems. This achieves electrical power saving without compromising the service quality.

## Figures and Tables

**Table 1:** Synthetic view of the On/Off, On/Idle and On/Off/Stag power optimization policies.

	Response time	Power consumption
On/Idle	Small response times	High waste of power
On/Off	Medium response times	Medium waste for low setup times, high for increasing setup
On/Off/Stag	Large response times	Low waste of power

**Table 2:** Server farm results for low *setuptime* (*E*[setup] = 1 sec).

*πδ*	*E*[*P*](*π*, *δ*) (W)		*E*[*T*](*π*, *δ*) (sec)		ERP(*π*, *δ*)	
	*ρ* ≤ 0.5	*ρ* → 1	*ρ* ≤ 0.5	*ρ* → 1	*ρ* ≤ 0.5	*ρ* → 1
On/Idle FIFO	6000	7100	1	1.8	6000	12780
On/Idle SPTF	6000	7100	1	1.3	6000	*9230 *
On/Off FIFO	4200	7100	1.2	2	**5040**	14200
On/off SPTF	4200	7100	1.2	1.35	**5040**	9585
